# The Association between Colorectal Cancer and Colonoscopic Conditions in Saudi Patients: A 10-Year Cross-Sectional-Retrospective Study

**DOI:** 10.4314/ejhs.v32i6.13

**Published:** 2022-11

**Authors:** Samah Alharbi, Arwa F Flemban, Saeed M Kabrah, Asim A Khogeer, Hanaa Alahmdi, Ahmed Abu Mansour, Adwa K Qari, Ghadi J Alqasimi, Lama S Almasoudi, Rahaf S Alotaibi, Raneem F Alskhairi, Rozan A AlHarbi

**Affiliations:** 1 Physiology Department, Faculty of Medicine, Umm Al-Qura University, Makkah, Kingdom of Saudi Arabia; 2 Pathology Department, Faculty of Medicine, Umm Al-Qura University, Makkah, Kingdom of Saudi Arabia; 3 Laboratory Medicine Department, Faculty of Applied Medical Sciences, Umm Al-Qura University, Makkah, Kingdom of Saudi Arabia; 4 Research Department, The Strategic Planning Administration, General Directorate of Health Affairs Makkah Region, Ministry of Health, Makkah, Kingdom of Saudi Arabia; 5 Medical Genetics Unit, Maternity & Children Hospital, Makkah Healthcare Cluster, Ministry of Health, Makkah, Kingdom of Saudi Arabia; 6 Faculty of Medicine, Umm Al-Qura University, Makkah, Kingdom of Saudi Arabia

**Keywords:** Colorectal Cancer, Saudi Arabia, Colonoscopy Screening, Polyp, Bleeding, Inflammation, Diverticulosis

## Abstract

**Background:**

In Saudi Arabia, colorectal cancer (CRC) is the most common cancer in males and the third most common cancer in females. The current gold standard for colorectal cancer diagnosis is colonoscopy. Several concerns regarding the balance of ordering colonoscopy procedures for patients presenting with signs and symptoms. There are also several concerns regarding over-ordering the procedure when unnecessary. The current study aimed to evaluate the association between colorectal cancer and colonoscopic conditions in Saudi patients.

**Methods:**

A 10-year cross-sectional study was conducted at Alnoor Specialty Hospital, Makkah, over the last ten years. Colonoscopy reports of patients were evaluated to identify the colonoscopy manifestations associated with mass, polyps, and bleeding.

**Results:**

The current study evaluated 2158 cases admitted to the hospital for colonoscopic diagnosis. Results indicated that most of the patients were males (55.4%). Additionally, results showed a significant statistical association between tumor and bleeding, polyp, and hemorrhage. Moreover, it highlighted the association between polyps and bleeding, inflammation, and diverticulosis.

**Conclusion:**

CRC screening in Saudi Arabia is comprehensive; however, there are a few areas for improvement, including standardization of colorectal cancer pathology reporting to improve the health system's quality. Also, the current study identified conditions that are significantly associated with reported colon polyps and tumors, which could aid in stratifying patients selected for screening via colonoscopy.

## Introduction

Colorectal cancer (CRC) is the third most common cancer worldwide, with an increasing incidence and mortality rate (1). The incidence has increased significantly in both genders over the past few decades (2, 3). In Saudi Arabia, CRC is the most common type of cancer in males and the third most common in females (less than 14 years old) (4, 5). According to the latest statistics published in 2018 by the Saudi Ministry of Health, the male-to-female ratio of CRC incidence is 115:100 (MOH, 2018). About 30% of CRC cases have distant metastases at diagnosis in Saudi Arabia, compared to only 10% in the United States (4, 6, 7). This raises the question of whether this is due to inadequate CRC screening programs or a lack of public knowledge or awareness of early signs of cancer.

Screening protocols for CRC vary internationally, including the newly approved blood test for methylated *Septin9*, stool testing, radiology, and colonoscopy procedures with a wide range of specificity and sensitivity (8–10). This is one of the obstacles to planning and implementing a successful screening of CRC globally (8, 11). Additionally, colonoscopy and other screening methods for CRC reduced mortality but did not reduce the incidence (12, 13). This urges the identification of CRC risk factors to help solidify the screening program strategies and improve patient selection criteria.

The management of colorectal cancer varies according to the disease stage and may include laparoscopic surgery, chemoradiotherapy, and sphincter-preserving surgery. Consequently, patients may suffer from malnutrition, bowel dysfunction, and urinary and sexual dysfunction. Follow-up care extended for three years and involved some serological tests and computed tomography (CT) scans to detect the recurrence of the disease (14).

Non-modifiable risk factors for CRC include age and genetic mutations of some genes, such as *APC, MLH1,* and *MSH2.* Westernized nutritional patterns, physical inactivity, obesity, and smoking were also considered risk factors for CRC (15, 16). The most identified CRC risk factors are inflammatory bowel diseases, including Crohn's disease and ulcerative colitis (17). Furthermore, active bleeding, diverticulosis, hemorrhoids, and a history of polyps are all considered CRC risk factors (18, 19).

Colorectal polyps were one of the most frequently reported conditions affecting the colon, but not all were considered to have malignant potential (20). Neoplastic polyps of the colon, however, are of clinical importance because they are considered premalignant conditions (21) and can be considered the earliest form of colorectal cancer CRC (10). Most polyps are usually detected in asymptomatic patients, requiring about ten years to acquire neoplastic and malignant properties. Removing polyps from the colon is imperative before gaining malignant properties (20). Winawer *et al.* concluded in their study that none of the asymptomatic patients that underwent polypectomy by colonoscopy died from CRC (22). This conclusion indicates the importance of early detection and removal of polyps, and colonoscopy remains the main procedure for identifying polyps. Other conditions are also considered risk factors for developing CRC, including inflammatory bowel disease (IBD), bleeding, and hemorrhoids.

Inflammatory bowel diseases (IBD) were epidemiologically highlighted as factors that increase the risk of CRC (17, 23). Other studies found that CRC is a major cause of death for cases diagnosed with IBD (8). This has been attributed to the repeated insults to the mucosa of the colon during the inflammatory episodes of the disease (8). The risk of developing CRC in IBD patients increases with age (17, 24). The relationship between IBD and CRC development has led many healthcare organizations in the United States and the United Kingdom to increase surveillance of the affected population (17). In fact, repeated colonoscopy in IBD patients resulted in early detection of CRC in IBD patients and reduced mortality consequently (17, 24, 25).

Previous studies recommend early colonoscopy for patients with gastrointestinal bleeding (26). It was found that around 93% of patients diagnosed with CRC presented with bleeding (21). Another study found that 77% of patients diagnosed with CRC had a positive fecal occult blood test (27). In 2013, a study found that CRC patients with symptomatic bleeding were less likely to have metastasis at diagnosis (28). This study signifies the importance of screening patients with colorectal bleeding as a symptom to reduce mortality if a malignancy causes it; therefore, early detection and intervention (26, 28).

Another factor that is related to the development of CRC is hemorrhoid. However, there are several debates regarding the association of this condition with CRC. A study conducted in Taiwan for ten years about the association between CRC and hemorrhoids concluded that patients with hemorrhoids had an increased risk of developing CRC (29). Other studies suggested that hemorrhoids give false-positive fecal occult blood tests, and therefore, they suggested excluding patients with hemorrhoids from CRC screening (30). This was contradicted by other studies that concluded that hemorrhoids delayed CRC diagnosis due to diagnostic processes other than colonoscopy (31, 32). Thus, emphasizing the importance of colonoscopy as a screening test for patients with hemorrhoids to avoid the missed opportunity of diagnosis.

Identifying the risk factors and/or early clinical signs associated with CRC facilitates the design of more effective and sensitive CRC screening programs. For that reason, identifying the relationship between different symptoms and clinical conditions in colonoscopy reports with the probability of having a mass and/or polyps was essential. This study aims to evaluate the association of these colonoscopy manifestations with identifying masses (tumors) and polyps.

## Methods

**Study design and population**: The current study is a 10-year cross-sectional retrospective analysis of patients who underwent colonoscopy procedures in Al Noor Specialist Hospital, Makkah, Saudi Arabia. It included an analysis of all colonoscopy reports between March 01, 2010, and December 30, 2020.

**Data collection**: The following data were collected from the Al Noor Specialist Hospital medical record archive: demographic data and complete colonoscopy reports. The colonoscopy reports were further evaluated to extract the selected conditions for analysis. These conditions included tumor and polyps, symptoms including bleeding, ulcers, inflammation, associated conditions (diverticulosis, hemorrhoids), and known risk factors (ulcerative colitis, Crohn's disease).

**Ethical approval**: The current study was approved by the Committee for Research Ethics for the Health of Makkah Region, Ministry of Health (H-02-K-076-0103-268).

**Statistical analysis**: The data analysis was conducted using the Statistical Packages for Social Sciences (SPSS) version 28 (IBM Corp, Armonk, NY, USA). Descriptive statistics of frequency and percentage and mean±standard deviation were performed for categorical and continuous variables, respectively. The chi-square test was used to examine differences in the prevalence of different categorical variables. A p-value ≤ 0.05 was considered statistically significant.

## Results

Frequency of reported conditions in colonoscopy reports: The current study included 2158 Saudi patients that underwent colonoscopy at Al Noor Specialist Hospital during the 2010–2020 period. These reports were evaluated, and several conditions were identified according to this study's methodology. Male patients were higher (55.4%) than female patients (44.6%). Out of the total number of patients, 8% and 14% of patients were reported to have tumors and polyps, respectively. Results showed that hemorrhoid and inflammation were the most reported conditions (41.4% and 17.3%, respectively). Additionally, bleeding, ulcers, and diverticulosis were reported. Ulcerative colitis and Crohn's disease were the least reported diseases, with only 2% for the former and 0.6% for the latter (Table 1).

**Table 1:** Characteristic of patients undergoing colonoscopy (n = 2158)

Characteristic		n	%
Total		2158	
	Female	963	44.6
Gender	Male	1195	55.4
Tumors	Yes	173	8.0
	No	1985	92.0
Polyp	Yes	313	14.5
	No	1845	85.5
Bleeding	Yes	249	11.5
	No	1909	88.5
Ulcer	Yes	223	10.3
	No	1935	89.7
Inflammation	Yes	373	17.3
	No	1784	82.7
Haemorrhoid	Yes	893	41.4
	No	1265	58.6
Diverticulosis	Yes	199	9.2
	No	1959	90.8
Ulcerative Colitis	Yes	44	2.0
	No	2114	98.0
Crohn's disease	Yes	12	0.6
	No	2146	99.4

Bleeding, polyp and hemorrhoid showed association with tumors in colonoscopy reports. Table 2 shows the association between tumors and gender, bleeding, polyp, ulcer, inflammation, diverticulosis, hemorrhoid, ulcerative colitis, and Crohn's disease. Results indicated that only 173 (8%) cases reported tumors. The association analysis indicated that the tumor was associated with a bleeding, polyp, and hemorrhoid (53, 43, and 36, respectively; *p*-value= 0.001). Results also indicated no statistical association between tumors and diverticulosis, ulcerative colitis, and Crohn's disease (11, 1, and 1, respectively; *p*-value > 0.005). Additionally, among the 173 cases, only 19 had an ulcer, and 21 had colorectal inflammation.

**Table 2:** The association of tumors with gender and other colonoscopic conditions

Characteristics		Tumour				Chi-Square	*p*-value
		YES		NO			

		n	%	n	%		
Gender	Female	82	3.8	881	40.8	0.586	0.444
	Male	91	4.2	1104	51.2		
Bleeding	Yes	53	2.5	196	9.1	67.202	0.001
	No	120	5.6	1789	82.9		
Polyp	Yes	43	2.0	270	12.5	16.251	0.001
	No	130	6.0	1715	79.5		
Ulcer	Yes	19	0.9	204	9.5	0.085	0.770
	No	154	7.1	1781	82.5		
Inflammation	Yes	21	1.0	352	16.3	3.493	0.062
	No	152	7.0	1632	75.6		
Diverticulosis	Yes	11	0.5	188	8.7	1.842	0.175
	No	162	7.5	1797	83.3		
Haemorrhoid	Yes	36	1.7	857	39.7	32.812	0.001
	No	137	6.3	1128	52.3		
Ulcerative Colitis	Yes	1	0.0	43	2.0	2.010	0.156
	No	172	8.0	1942	90.0		
Crohn's disease	Yes	1	0.0	11	0.5	0.002	0.968
	No	172	8.0	1974	91.5		

The impact of the clinical manifestation of CRC on cancer diagnosis: The association of polyp with gender, bleeding, ulcer, inflammation, diverticulosis, hemorrhoid, ulcerative colitis, and Crohn's disease are presented in table 3. Results indicated that 313 patients (14.5%) reported having polyp in their colonoscopy report. Furthermore, polyps' formation was higher in male than female patients (203 and 110, respectively) with a highly significant difference (*p*-value= 0.001). Inflammation, bleeding, and diverticulosis were polyps' most common associated conditions (71, 49, and 40 cases, respectively). The association between polyp and inflammation was highly statistically significant (*p*-value= 0.006). The association between reporting polyps with bleeding and diverticulosis was also significant (*p*-value= 0.014 and 0.019, respectively).

**Table 3:** The association of the polyp with gender and other colonoscopic conditions

Characteristics		Polyp				Chi-Square	*p*-value

		YES		NO			
		n	%	n	%		
Gender	Female	110	5.1	853	39.5	13.317	0.001
	Male	203	9.4	992	46.0		
Bleeding	Yes	49	2.3	200	9.3	6.078	0.014
	No	264	12.2	1645	76.2		
Ulcer	Yes	38	1.8	185	8.6	1.290	0.256
	No	275	12.7	1660	76.9		
Inflammation	Yes	71	3.3	302	14.0	7.440	0.006
	No	242	11.2	1542	71.5		
Diverticulosis	Yes	40	1.9	159	7.4	5.537	0.019
	No	273	12.7	1686	78.1		
Haemorrhoid	Yes	130	6.0	763	35.4	0.004	0.953
	No	183	8.5	1082	50.1		
Ulcerative Colitis	Yes	7	0.3	37	1.7	0.071	0.789
	No	306	14.2	1808	83.8		
Crohn's disease	Yes	1	0.0	11	0.5	0.371	0.543
	No	312	14.5	1834	85.0		

## Discussion

The present study investigated the colonoscopic reports of patients admitted to Alnoor Specialty Hospital over the last ten years. Results revealed that most patients who underwent colonoscopy were male, and the main reported conditions were hemorrhoids, polyps, and inflammation. Although colon cancer is more common in males, other colonic disorders such as colonic polyps, Crohn's disease, and hemorrhoids are more frequent in females (33–35). Thus, gender-specific differences in colonic disorders should be acknowledged in colonoscopy testing/screening reports to avoid late diagnosis and improve the management and prevent deterioration of the case.

The findings of the current study showed that colonic tumor was significantly associated with bleeding, polyps, and hemorrhoids. At the same time, colonic polyps were significantly associated with inflammation, diverticulosis, and bleeding. Although only 2.3% of patients with polyps and 2.5% with tumors presented with bleeding, it was considered a significant factor related to colonic conditions, tumors, and polyps. Bleeding is one of the most common symptoms of colorectal polyps and indicates colonoscopy (36). Moreover, it was considered an acute complication in patients with colon cancer (37). Bleeding was also associated with various colon ulcerative, benign, and malignant conditions (38). Another study demonstrated that bleeding was the main symptom in most CRC patients (28). Collectively, these findings highlight the necessity of colonoscopy for patients with colonic bleeding.

Similar to previously published findings, our data identified a significant association between colonic tumors and hemorrhoids. A study has suggested that patients with hemorrhoids had a roughly two-fold greater chance of developing CRC and highlighted the urge to screen colonoscopy for patients with hemorrhoids (29). Furthermore, colonoscopy was recommended for patients with hemorrhoids because CRC and hemorrhoids share similar lifestyle-associated risk factors, including low fiber consumption, obesity, and insufficient exercise (39).

Although polyps were considered benign growth, polypectomy was essential to CRC prevention (40). Therefore, patients with a history of polyps are subject to annual surveillance colonoscopy (41). Another benign colonic condition that was found to be associated with polyps was diverticulosis. Several studies investigated diverticulosis as a risk factor for polyps and/or CRC and showed conflicting findings. Some studies argue that diverticulitis was not a risk factor for polyps or CRC (42, 43). Several studies showed a significant association between diverticulosis and pre-cancerous polyps, but patients with diverticulosis were highly likely to develop polyps (25, 44, 45). Other studies have also linked diverticulosis to CRC (46, 47). Extensive prospective cohort studies were needed to validate the conflicting data on diverticular disease as a risk factor to determine the significance of colonoscopy following the diagnosis of diverticular disease by ultrasound abdominal ultrasound and elevated C-reactive protein (CRP) value.

The current data showed that inflammation was significantly associated with polyps but not tumors. However, the association between inflammation and CRC was well established (48). Colonic inflammation includes colitis, salmonellosis, shigellosis, travelers' diarrhea, diverticular disease, inflammatory bowel syndrome, and inflammatory bowel disease. Colonoscopy reports evaluated in the current cohort study lacked histopathological findings, including the classification of the inflammatory conditions. This limitation complicates the analysis of the association between polyps and different types of colonic inflammation in the current data set. However, previous reports showed multiple associations between colonic inflammatory conditions (49). Inflammatory colon conditions, such as microscopic colitis, inflammatory bowel disease (IBD), and active colitis, are associated with decreased prevalence of all types of colon polyps, including hyperplastic polyps, serrated adenomas, and adenomas (50, 51). In contrast, IBD, including Ulcerative Colitis and Crohn's disease, were significantly associated with tumor development (17, 49).

Still, several challenges were under review to follow up cases of history of polyps, and several researchers were working on improving the recommendations for post-polypectomy surveillance by colonoscopy (24). The problem with post-polypectomy screening is that patients are usually asymptomatic, which leads to a drain on the healthcare system. Also, increased intervals between screenings could result in missed diagnoses in some cases (24). Altogether, this signifies the importance of conducting more analyses of retrospective studies to improve the guidelines and recommendations for post-polypectomy surveillance based on evidence.

Ideal guidelines for colonoscopy ought to lead to early detection of CRC, improve prognosis, and effectively use medical resources and personnel. Early detection of CRC was associated with a better prognosis and reduced mortality from CRC (52, 53). Current screening guidelines involve tests to detect the presence of blood in the stool, such as fecal occult blood tests and fecal immunochemical tests. CRC screening guidelines also involve colonoscopy or sigmoidoscopy but less frequently and at an older age (beginning at age 50) (54). With the increased incidence and morbidity from CRC, screening guidelines should be revisited, especially because studies showed that screening tests for fecal blood were ineffective (55), and colonoscopy might miss some CRC lesions. Therefore, symptoms and clinical conditions should be identified, and their association with CRC investigated to develop well-detailed guidelines for screening and surveillance colonoscopy.

A large-scale study is not available in Saudi Arabia and is necessary to evaluate the colonoscopic conditions diagnosed during the procedure. The current study compromises a sample collected from a single hospital in Makkah. The sample size included more than 2000 patients admitted to Alnoor Specialty Hospital for ten years. Though a higher number of male patients underwent colonoscopy, there was a significant association of polyps in female patients. In the same sample, there was a relationship between identifying tumors and other conditions, including bleeding, polyps, and hemorrhoids.

On the other hand, polyp identification in patients was associated with bleeding, inflammation, and diverticulosis. The research has previously identified such an association, and the current study found that the conditions correlated with tumors are somewhat different from those correlated with polyp identification. This kind of study aids in identifying patients with high risk and aids in stratifying them for early screening of colorectal cancer. Until this study was conducted, few publications reviewed the retrospective data on colonoscopy in Saudi Arabia. Additional research on a larger dataset collected from different centers is recommended to verify this study's conclusion and draw a nationwide conclusion.

In conclusion, this study shows that CRC screening in Saudi Arabia is comprehensive; however, there are few rooms for improvement, such as standardization of colorectal cancer pathology reporting. Quality pathology reporting could improve cancer management as well as facilitate international comparisons. Also, more efforts should be made to improve public awareness about the risk factors of CRC and the importance of colonoscopy screening.
